# Original Communication

**Published:** 1841-06-12

**Authors:** J. C. Mercer

**Affiliations:** Williamsburg


### MEDICAL EXAMINER.

DEVOTED TO MEDICINE, SURGERY, AND THE COLLATERAL SCIENCES.

No. 24.] PHILADELPHIA, SATURDAY, JUNE 12, 1841. [Vol. IV.

### ORIGINAL COMMUNICATION.

To the Editors of the Medical Examiner.

*Gentlemen*—In your remarks, appended to my late communication on the subject of an extraordinary case of tubercular disease, you observe, "It is only to be regretted that the author does not describe more fully the nature of the lesions of the tricuspid valve, and the condition of the rest of the heart, &c." Now, in justice to myself,—as well as for your own satisfaction and that of your readers, it should be stated, that the "rest of the heart" exhibited no departure whatever from the healthy standard. It was presumed that my silence on this head would be a sufficient index of the fact, that the lining and enveloping membranes, and muscular structure of the organ, were altogether natural. The functions of the diseased valve were entirely impeded by its conversion into a mass of tuberculous matter, very destructible under the slightest pressure of the fingers.

The history of the case should have embraced the fact, that, before parturition, no complaints were made as to any difficulty about the heart or the respiratory organs. But in two or three days after labour had taken place, the symptoms before described—consisting of irregular chills, followed by fever and sweating,—occurred, and terminated fatally in about a fortnight.

I by no means, gentlemen, intend to intimate that "the tendency to tuberculous disease, and the simultaneous deposit of granulations in the serous membranes and the lungs" are not well known facts. I merely design saying that tubercles are the consequence, and not the cause, of the peculiar disturbance accompanying them, and that they are dependent on the *tubercular diathesis*, contrary to the opinion that has been maintained, of their being exclusively the result of inflammation.

Do you not regard the prevalence of irregular chills, succeeded by fever and sweating, as a species of hectic? In the case under consideration the symptoms, as far as these three go, were those of the more advanced stages of phthisis.

Can you allow the spirit of these observations to appear in your periodical, and will you gratify me by responding to the question propounded above, as it is asked for the sake of information?

With respect,

Your obedient servant,

J. C. Mercer.

*Williamsburg, May 1st*, 1841.

The statement of our correspondent in regard to the condition of the rest of the heart, makes the case much more conclusive and satisfactory. In reports of this kind, silence does not prove that the organ alluded to is in a normal condition; it is then only probable that no important alteration is present.

The class of cases to which this belongs, undoubtedly affords a convincing proof of the constitutional origin of many cases of phthisis, and show that the tubercles are a consequence of a general disorder. There are even cases in which the exciting cause of tubercles seems altogether local, but is still dependant upon the constitutional diathesis; but the action of a general cause is not so evident here as in those instances in which a simultaneous tuberculous deposit occurs in different organs. The opinion that tubercles are exclusively the result of inflammation, is of late years maintained by very few individuals.

The cases of very extensive tuberculous disease, and of tuberculous pleurisy, are generally acute and rapid in their progress. In all of them the irritative fever is extremely high, and is a *species* of hectic, but not the true hectic; that is, it is much less regular in its course, and the chills are less severe.

The fever of acute phthisis varies very much in intensity; it is sometimes extremely violent, with a very frequent and irritated pulse; in other cases the pulse is quick and corded, but less frequent, and nearly similar to that of pleurisy. In fact, the close analogy of the fever in these cases is one of the points of resemblance between some cases of pleurisy and phthisis.

